# Crystal structure of the human Tip41 orthologue, TIPRL, reveals a novel fold and a binding site for the PP2Ac C-terminus

**DOI:** 10.1038/srep30813

**Published:** 2016-08-04

**Authors:** Valéria Scorsato, Tatiani B. Lima, Germanna L. Righetto, Nilson I. T. Zanchin, José Brandão-Neto, James Sandy, Humberto D’Muniz Pereira, Állan J. R. Ferrari, Fabio C. Gozzo, Juliana H. C. Smetana, Ricardo Aparicio

**Affiliations:** 1 Institute of Chemistry, University of Campinas, Campinas, São Paulo 13083-970, Brazil; 2 Brazilian National Laboratory for Biosciences, Center for Research in Energy and Materials, Campinas, São Paulo 13084-971, Brazil; 3 Institute of Biology, University of Campinas, Campinas, São Paulo 13083-970, Brazil; 4 Carlos Chagas Insitute, Oswaldo Cruz Foundation (FIOCRUZ), Curitiba, P.R., Brazil; 5 Diamond Light Source, OX11 0DE, Chilton, UK; 6 Physics Institute of São Carlos, University of São Paulo, São Carlos, São Paulo 13563-120, Brazil

## Abstract

TOR signaling pathway regulator-like (TIPRL) is a regulatory protein which inhibits the catalytic subunits of Type 2A phosphatases. Several cellular contexts have been proposed for TIPRL, such as regulation of mTOR signaling, inhibition of apoptosis and biogenesis and recycling of PP2A, however, the underlying molecular mechanism is still poorly understood. We have solved the crystal structure of human TIPRL at 2.15 Å resolution. The structure is a novel fold organized around a central core of antiparallel beta-sheet, showing an N-terminal α/β region at one of its surfaces and a conserved cleft at the opposite surface. Inside this cleft, we found a peptide derived from TEV-mediated cleavage of the affinity tag. We show by mutagenesis, pulldown and hydrogen/deuterium exchange mass spectrometry that this peptide is a mimic for the conserved C-terminal tail of PP2A, an important region of the phosphatase which regulates holoenzyme assembly, and TIPRL preferentially binds the unmodified version of the PP2A-tail mimetic peptide DYFL compared to its tyrosine-phosphorylated version. A docking model of the TIPRL-PP2Ac complex suggests that TIPRL blocks the phosphatase’s active site, providing a structural framework for the function of TIPRL in PP2A inhibition.

Reversible phosphorylation orchestrates most signaling pathways. Type 2A phosphatases, comprising PP2A, PP4 and PP6, are serine/threonine phosphatases of the PPP subfamily. These phosphatases are structurally and evolutionarily related to Protein Phosphatase 1 (PP1). They share particular distinctive characteristics such as sensitivity to inhibition by okadaic acid in the nanomolar range and the presence of a conserved C-terminal motif (PDYFL) that is subject to post-translational regulation in PP2Ac^1^,^2^ and possibly also in PP4c and PP6c^3^. PP2A is a major regulator of several cellular processes and an important tumor suppressor. Its functional unit is a heterotrimeric holoenzyme composed of a catalytic subunit (PP2Ac), a scaffold subunit (PR65/A) and a regulatory subunit which might belong to four structurally diverse families (B, B′, B″ and striatins)^4^,^5^,^6^. The selectivity is mediated by post-translational modifications of the C-terminal tail, which can be phosphorylated on the tyrosine residue or methylated on the extreme C-terminal leucine^7^,^8^.

The biogenesis of PP2A, defined as the process undergone by the phosphatase catalytic subunit from translation of the polypeptide to its incorporation into functional holoenzymes, is regulated by a series of steps which include stabilization of a latent, inactive form by immunoglobulin binding protein 1 (IGPB1)/α4 protein^9^, ATP-dependent transfer of catalytic metal ions by PTPA (Phosphotyrosyl Phosphatase Activator)^10^ and subsequent methylation of the C-terminal leucine (Leu309) by LCMT-1 (Leucine Carboxy Methyl Transferase)^11^. In a recent review, Sents *et al*. proposed that five proteins are responsible for the biogenesis of PP2A: LCMT1, PME-1, PTPA, α4 and potentially TIPRL^4^.

TIPRL (TIP41, TOR signaling pathway regulator-like) is a type 2A phosphatase regulatory protein which is still poorly understood both structurally and functionally. This is in part due to the lack of any structural information which may guide further studies of its structure-function relationship. In a previous study, we performed a yeast two-hybrid screen using human TIPRL as bait and found that it binds the catalytic subunits of PP2A, PP4 and PP6 and inhibits the activity of PP2A *in vitro*^12^. A recent study showed that TIPRL is overexpressed in hepatocellular carcinoma and its down-regulation synergizes with TRAIL to induce apoptotic death of tumor cells^13^. TIPRL has been considered a part of the mTOR signaling because of the function therein of its yeast counterpart, Tip41^14^, but experimental evidence was lacking until recently. However, contrary to the yeast Tip41, which inhibits TOR signaling by sequestering the α4 orthologue Tap42, human TIPRL positively regulates mTORC1 signaling in response to amino acids^15^.

During the past decade, the mechanism of PP2A regulation was unraveled via a series of crystal structures of its regulatory proteins, either isolated^16^,^17^,^18^,^19^,^20^ or in complex with PP2Ac or the PP2A core enzyme^9^,^10^,^11^,^21^,^22^. However, TIPRL was still eluding most efforts and remained largely uncharacterized. Here, we report the crystal structure of human TIPRL, revealing a novel protein fold bound to a TEV site-derived peptide which mimics the conserved C-terminus of PP2Ac. Our findings provide a structural framework to interpret the inhibition of PP2A by TIPRL.

## Results

### Overall structure of TIPRL

After extensive crystallization attempts using full length TIPRL without success, we decided to use a short N-terminal deletion to eliminate potential flexible regions. The structure of this shortened protein, hereafter named TIPRLΔN, was determined by selenomethionine-SAD.

Data collection and refinement statistics are shown in Table 1. The structure obtained through selenomethionine experimental phasing consists of a new fold of α-helices and 3_10_ helices organized around a beta-sandwich core in a unique topology. The last 16 C-terminal residues of TIPRL show no electron density in the crystal structure, indicating structural disorder. Structural similarity searches on DALI^23^, Top Search and CATH^24^ did not find significant hits. For CATH analyses, the closest match is the 3tutA02 domain, with a SSAP score of 70 and an RMSD of 6.5 Å. Visual inspection of 3tutA02 domain reveals that the number and relative orientation of secondary structure elements are very different from TIPRL structure.

**Figure 1:**
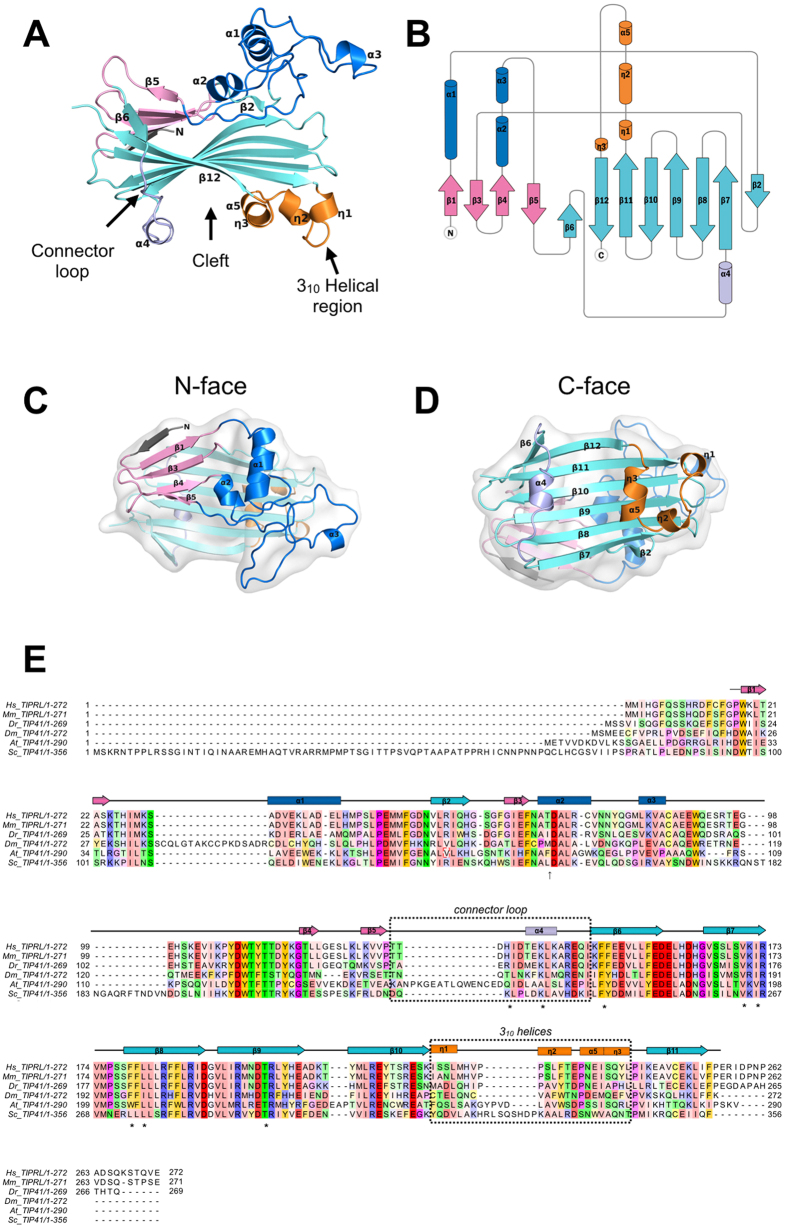
Overall structure of TIPRL. (**A**) Cartoon representation of the TIPRL fold highlighting its structural elements. (**B**) Topology diagram of the TIPRL fold generated with TopDraw. (**C**) Top and (**D**) bottom views of the cartoon representation combined with transparency representation of the surface where the secondary structure elements are labelled form N- to C-terminus. (**E**) The sequence conservation pattern of TIPRL/TIP41. The amino acid sequences of TIPRL from *Homo sapiens* (Hs) and *Mus musculus* (Mm) and TIP41 from *Danio rerio* (Dr), *Drosophila melanogaster* (Dm), *Arabidopsis thaliana* (At) and *Saccharomyces cerevisiae* (Sc) were aligned using ClustalW and color coded according to their degree of evolutionary conservation and physicochemical properties using the color scheme Zappo 21 from Jalview (aliphatic/hydrophobic: salmon, aromatic: orange, positive: blue, negative: red, hydrophilic: green, conformationally special: pink, cysteine: yellow). The secondary structure elements from the human TIPRLΔN structure are represented on top of the alignment. Arrows represent beta strands, rectangles represent α-helices and 3_10_ helices as indicated. The color scheme of the secondary structure elements follows the other panels.

**Figure 2:**
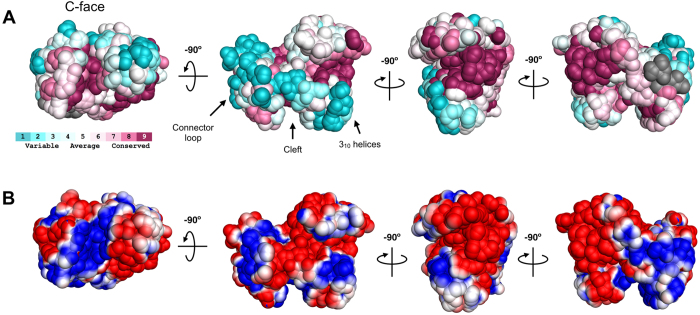
Surface properties of TIPRL. (**A**) The sequence conservation pattern of TIPRL was obtained using the Consurf server. The surface representation is colored from the least conserved (turquoise) through intermediately conserved positions (white) to the most conserved residues (burgundy). (**B**) Surface representation of the macromolecular electrostatics calculation by the PyMol APBS (Adaptive Poisson-Boltzmann Solver). The second image of each series (from left to right) is in the same orientation as Fig. 1A.

The TIPRL structure is organized around an antiparallel beta-sheet core composed by six long strands of up to 13 residues. This sheet is covered by a region consisting of a smaller five strand beta-sheet along with two alpha-helices and a considerable amount of residues that adopt non-repetitive structures (Fig. 1). This region corresponds to the N-terminus, while the beta-sheet core corresponds to the C-terminus and consists mostly of conserved residues among TIPRL orthologs. Both N- and C-terminal regions are connected through a long loop (residues 121–148) containing a short segment in alpha-helical conformation, which embraces the beta sheet core and forms a cleft along with a small, helical region located opposite to it, predominantly formed by 3_10_ helices. For the purpose of further discussion, we define the cleft side as the C-face, the N-terminal region as the N-face (Fig. 1C,D), and residues 121–148 as the connector loop. The C-terminal beta-sheet core shows the highest evolutionary conservation, highlighting its structural importance (Figs 1E and 2A). The electrostatic potential shows a predominantly negative surface while the cleft concentrates positively charged residues which are also highly conserved (Fig. 2B).

### TIPRLΔN crystallizes as a dimer but TIPRL is likely a monomer in the cell

The asymmetric unit of the crystal contained two molecules that seem to form a dimer. Both molecules were refined without applying NCS restraints independently, and do not show any major differences (all-atom RMSD value of 0.3 obtained with PyMol Align tool). Interestingly, a dimer was observed in solution during the purification of TIPRLΔN, but not full length TIPRL. Additionally, TIPRLΔN, but not full length TIPRL, was able to dimerize only after proteolytic removal of the affinity tag, which was usually incomplete resulting in a double band on SDS-PAGE analysis (Fig. S1). Despite its apparent heterogeneity, this preparation consistently crystallized, while several other preparations that were not subjected to TEV cleavage failed to do so. Analysis of size exclusion profiles under different conditions allowed us to conclude that dimerization occurs only if these three conditions are satisfied: 1- deletion of the N-terminal residues, 2- cleavage of the histidine tag, and 3- high concentration (Fig. S1B). Crystals were formed from highly purified preparations of cleaved, dimeric protein as well as mixed fractions containing both cleaved and uncleaved protein.

The β-strand in the N-terminus of TIPRLΔN (β1) is critical to form the dimer interface in the asymmetric unit and its amino acid residues originate from a plasmid-derived sequence instead of TIPRL itself (GHMAS), agreeing with the fact that the full length TIPRL construct does not dimerize (Fig. S2A). The preformed dimer appears to be a necessary requisite for nucleation instead of an artifact produced by the crystallization conditions. However, due to the conditions described above that must be fulfilled to allow its appearance, it is unlikely to be physiologically relevant.

### Structure in solution

Chemical (lysine) cross-linking/mass spectrometry experiments were performed to provide information about the solution structure of TIPRL, and in particular dimer formation and the conformational flexibility of the C-terminus (Table S1). Evidence for the proposed dimer comes from the observation of an interchain, symmetrical cross-link in a peptide from the N-terminal region, _25_THIMKSADVEK_35_. In the crystal structure, the distance between the alpha carbons of K29 in the presumed dimer is 18.4 Å and the NZ distance is 24.4 Å, which can be brought within the DSS reach (13.5 Å) with small changes in the conformation of both Lys side chains (Fig. S2B).

The tryptic peptide corresponding to the C-terminus of TIPRL, _258_IDPNPADSQKSTQVE_271_, was found crosslinked to eight different peptides that span the primary sequence from N- to C-terminus: _12_DFCFGPWKLTASK_24_, _25_THIMKSADVEK_35_, _30_SADVEKLADELHMPSLPEMMFGDNVLR_56_, _118_GTLLGESLKLK_128_, _127_LKVVPTTDHIDTEK_140_, _141_LKAR_144_, _201_LYHEADKTYMLR_212_ and _246_EAVCEKLIFPER_257_. All of them contain a lysine that is located within 20 Å of the C-terminus (Fig. S3). The exceptions are K29 and K142, which are located in opposite sides on the TIPRL surface (the N-face and the connector loop, respectively). This variety in cross-linking agrees with a high flexibility of this C-terminal segment. Although this short segment is not conserved, it harbors two experimentally validated phosphorylation sites. S265 was detected along with S239 as a phosphorylation site for ATM/ATR kinases^25^ and S268 is present in PHOSIDA as an observed phosphorylated site from mouse brain samples. All remaining identified cross-links are in agreement with the crystal structure indicating that with the exception of the C-terminus, the protein behaves essentially as a rigid domain.

### Identification of the binding site for the C-terminus of PP2Ac

**Figure 3:**
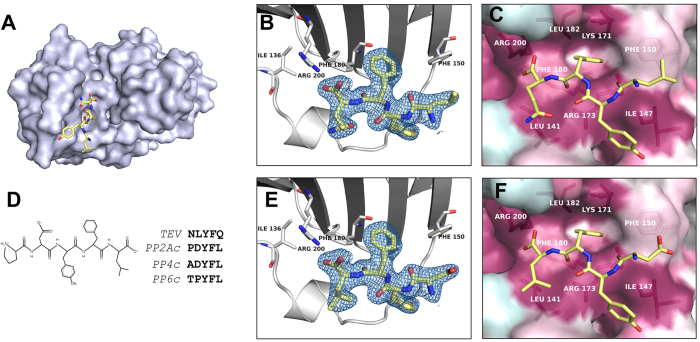
A conserved peptide binding site specifically recognizes the C-terminus of PP2Ac. (**A**) Overview of the last four residues of the peptide generated after cleavage with the TEV protease (MGHHHHHHENLYFQ), LYFQ, bound to TIPRL (chain A). The peptide fragment is depicted as sticks over a surface representation of TIPRL, viewed from the bottom. (**B**) Close up view of the TEV site peptide LYFQ fitted into the electron density map (2*Fobs*–*Fcalc*) depicted at 1.0 σ. (**C**) Conservation pattern of the peptide binding site showing the LYFQ peptide in sticks representation. Consurf conservation scores mapped on the surface are colored as in Fig. 2 (burgundy: more conserved; turquoise: less conserved) and relevant amino acid side chains are labelled underneath the surface representation. (**D**) Sequence similarity between the TEV site peptide and the conserved C-terminus of type 2A phosphatases (PP2Ac, PP4c and PP6c). (**E**) The C-terminus of PP2Ac, DYFL, was fitted into the electron density map (2*Fobs*–*Fcalc*) of the LYFQ peptide depicted at 1.0 σ. (**F**) Same representation as Fig. 4C, showing the PP2Ac-derived peptide DYFL instead of TEV peptide.

The density maps of both molecules in the asymmetric unit present a positive electron density inside the bottom cleft that cannot be accounted by the TIPRL sequence. This positive density can be correctly modeled as the four C-terminal residues from the TEV cleavage site, LYFQ, bound to the TIPRL chain A (Fig. 3). The same peptide was bound to the symmetric binding site on chain B and stabilized by further interactions with chain A, which allowed for the modelling of the entire TEV site, ENLYFQ. This finding implied that the peptide generated after cleavage with the TEV protease (MGHHHHHHENLYFQ) shows a high affinity for the TIPRL cleft in the C-face, which allows it to stay tightly bound to the protein during the size exclusion and buffer exchange steps instead of being lost by diffusion. Most of the N-terminal region of this peptide is intrinsically disordered, pointing towards a solvent-filled channel of the crystal and only the C-terminal four to six amino acids are seen bound to TIPRL.

The binding site for the peptide shows alternating patches of hydrophobic (I147/F150 and L141/L182) and positively charged residues (K171/R173 and R184/R200). Most of the TIPRL-peptide interface is stabilized by hydrophobic interactions. One major exception is R200, which engages in ionic interactions with the C-terminal carboxylate group. These residues of TIPRL interacting with the peptide are highly conserved among type 2A phosphatases, suggesting a biologically relevant, functional binding site rather than a spurious artifact (Fig. 3C). Upon careful examination of the TEV recognition site, we found that its sequence resembles the conserved C-terminus of PP2Ac, which is also shared by the related phosphatases PP4 and PP6 (Fig. 3D). Indeed, both sequences – LYFQ from the TEV site and DYFL from the PP2Ac C-terminus – would fit into the binding site without steric clashes, allowing the latter to be modeled inside the electron density (Fig. 3E,F). By analyzing this structural model, it can be easily seen that phosphorylation on Y307 would result in steric hindrance, while methylation of the carboxylate group of the C-terminus could be easily accommodated. These observations raised an intriguing possibility for the regulation of PP2Ac binding to TIPRL.

As reported recently^15^, TIPRL preferentially binds methylated PP2Ac and the mutation Y307E on PP2Ac prevents its interaction with TIPRL, which the authors interpreted as evidence for regulation based on the methylation status of the C-terminus. Based on our structural analysis, it is more likely that this mutation directly affects the interaction rather than indirectly interfering with the methylation of L309.

### Mutational analysis of the TIPRL-PP2Ac interaction and HDX/MS

**Figure 4:**
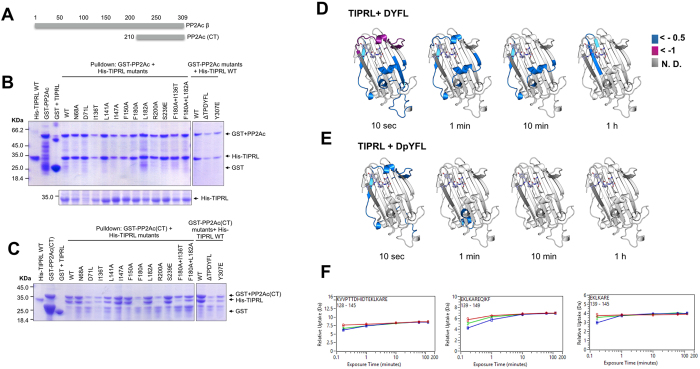
Validation of the PP2A C-terminus binding site on TIPRL by mutagenesis/pulldown and HDX/MS. (**A**) Schematic representation of the PP2A constructs used for GST pulldown. (**B**) GST-pulldown assays of mutated TIPRL. GST-tagged, full length PP2Acβ and His-tagged, full length TIPRL or its mutated versions were co-expressed in *E. coli* and the lysates were incubated with glutathione-sepharose beads. The right panel shows WT His-TIPRL combined with a truncation mutation (ΔTPDYFL) and a phosphomimetic mutation of PP2Ac (Y307E). No substantial effects were observed for any of the mutations. The lower panel depicts the soluble lysates, showing reduced expression levels for D71L. (**C**) The TIPRL mutants in (**B**) were assayed for their interaction with PP2Ac (residues 210–309). The mutants R200A and F180A + I136T show substantial loss of binding to PP2Ac. (**D**,**E**) Structural representation of the relative deuterium incorporation (in comparison with apo protein) from HDX-MS analysis of full length, his-tagged TIPRL, in the presence of a synthetic tetrapeptide which mimics the PP2A C-terminus, unmodified (**D**) or phosphorylated (**E**). The bottom side of TIPRL is shown in cartoon representation and the DYFL peptide is represented as blue sticks. The relative deuterium exchange is represented as follows: Blue: 0 to −0.5 Da, Purple: 0.5 to −1.0 Da, Dark grey: not determined. In both cases, only negative values were observed, indicating either a gain of structure or reduced solvent exposure compared to the control sample with no bound peptide. The 2 hour time points are identical to the respective 1 hour timepoint and therefore are not depicted in the figure. (**F**) Representative deuterium incorporation plots of peptides from the TIPRL connector loop, in the apo protein (red) or in the presence of a synthetic tetrapeptide which mimics the PP2A C-terminus, unmodified (blue) or phosphorylated (green).

We used a combined approach of mutational analyses and hydrogen-deuterium exchange/mass spectrometry to validate the PP2Ac binding site on TIPRL and to explore the potential role played by the post-translational modifications in the regulation of this interaction (Fig. 4).

Our previous studies identified mutations in the sequence of TIPRL that impaired its interaction with the catalytic subunits of type 2A phosphatases (including PP2Ac, PP4c and PP6c) using a random, unbiased reverse two-hybrid screen^12^. The mutants D71L, I136T, T138S, M196V and D198N were deficient in phosphatase binding as measured by a yeast two-hybrid assay. In the same study, we also showed that residues 210–309 of PP2Ac were sufficient to bind TIPRL both in the yeast two-hybrid and in GST pulldown assays. Interestingly, residues I136, T138, M196 and D198 are all close to the peptide binding site, which strongly supports our hypothesis that this site is involved in the interaction with PP2Ac. D71 is located in an opposite region, as discussed above.

We selected two mutants from our previous study (D71L and I136T) and designed mutations in other residues based on their sequence conservation and presence in the putative PP2Ac binding site. Residue N68, which is conserved and close to D71, was also mutated. The ability of these mutations to disrupt the interaction with either full-length or N-terminally truncated PP2Ac (residues 210–309) was assessed in a GST-pulldown assay using recombinant proteins co-expressed in *E. coli* (Fig. 4B,C). Intriguingly, the interaction with full length PP2Ac could not be disrupted by any of the TIPRL mutations, while mutants F180A/I136T, and R200A showed remarkably impaired binding to the truncated version of PP2Ac (residues 210–309). This finding suggests an extensive TIPRL-PP2Ac interaction surface which includes the extreme C-terminus of PP2Ac but is not restricted to it. Most likely, upon deletion of a major part of the PP2Ac sequence, the interaction of TIPRL with the remaining PP2Ac fragment is stabilized by contacts with the extreme C-terminus.

Conversely, we modified the extreme C-terminus of PP2Ac by deleting the last five residues or changing Y307 to Glutamate (Y307E), both on full length and C-terminal (210–309) constructs, and assessed the interaction with wild type TIPRL by GST pulldown. These mutations had no appreciable effect on the interaction (Fig. 4B,C).

Additional insight was obtained from HDX/MS experiments in which purified TIPRL was incubated with the synthetic DYFL peptide, either phosphorylated on the tyrosine residue or unmodified. Compared to the apo protein, these four residues which mimic the four C-terminal residues of PP2Ac were sufficient to cause appreciable reduction of backbone amide deuterium uptake on TIPRL. The most prominent change was the reduction of deuterium uptake by helix α4 from the connector loop that directly interacts with the peptide in the crystal structure, thus decreasing the solvent exposure upon binding. HDX/MS data also reveals a decrease in deuterium uptake in the 3_10_ helices η2 and η3 and helix α5 (Fig. 4D). This region is not directly connected to the ligand peptide and shows that upon ligand binding, this region becomes more structured (less flexible) as a secondary effect, in agreement with the fact that TIPRL does not crystallize in the absence of the ligand. Additionally, HDX/MS data from the experiment performed with phosphorylated peptide (DpYFL) show the same trends but with a much reduced magnitude (Fig. 4E), indicating that the affinity of TIPRL for the phosphorylated peptide is much lower than the non-phosphorylated one in agreement with analysis of the crystal structure.

Taken together, the mutagenesis/pulldown and HDX experiments suggest that the extreme C-terminus of PP2Ac is sufficient, but not necessary to bind TIPRL, and support the hypothesis that the binding site for TEV-derived peptide in the crystal structure is actually the binding site of PP2A C-terminus.

### Structural model of the TIPRL-PP2Ac interaction

**Figure 5:**
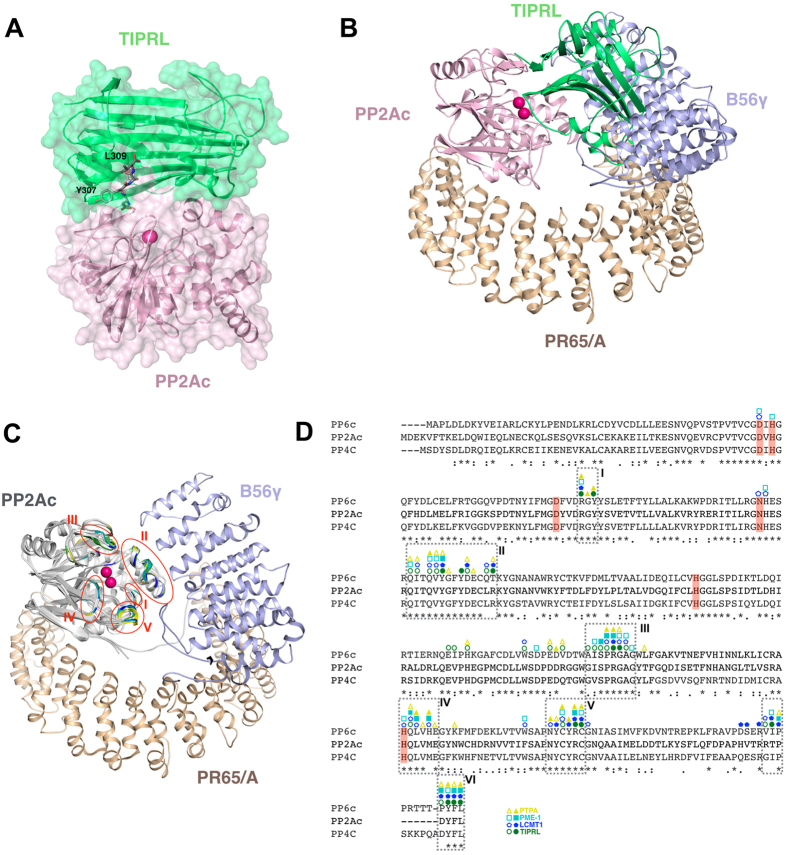
Structural model of the TIPRL-PP2Ac interaction. (**A**) Overall view of the TIPRL-PP2Ac complex, integrating information from the final docking model (PP2Ac residues 1–298, shown in cartoon/surface representation) and the crystal structure (PP2Ac residues 306–309, in sticks representation). The flexible residues (299–305) which were built manually in the docking model are not shown in the figure. (**B**) Superposition of the docking model of the TIPRL-PP2Ac complex to the PP2A trimeric complex (PDB: 2IAE chain A,B) showing partial overlap of TIPRL and B56γ. (**C**) The overlapping binding regions for LCMT1 (blue), PME-1 (cyan) PTPA (yellow) and TIPRL (green) are represented on the structure of the PP2A trimeric complex (PDB: 2IAE). The binding regions numbered I–V were mapped by a Pymol script using PDB entries 3P71, 3C5W, 4LAC and the TIPRL-PP2Ac docking model, respectively. Region II includes the helix switch (residues 120–126)^9^. The PP2Ac C-terminal region is shown only for the trimeric structure (PDB:2IAE). (**D**) Sequence alignment of the type 2A phosphatases PP2Ac, PP4c and PP6c, obtained with ClustalW, showing the binding regions for LCMT1, PME-1, PTPA and TIPRL as represented in Fig. 5C. Each complex was analysed using the Protein interfaces, surfaces and assemblies’ service PISA at the European Bioinformatics Institute^9^ (http://www.ebi.ac.uk/pdbe/prot_int/pistart.html), to detect amino acid residues engaged in hydrogen bonds or salt bridges (represented by full symbols) and those present on the interface but not directly involved in interactions (represented by empty symbols). The PP2Ac active site residues engaged in catalytic metal ion chelation are highlighted in red.

To build a model of TIPRL bound to full length PP2Ac, we hypothesized that there is a stable TIPRL-PP2Ac interface independent from the peptide binding cleft, as the mutagenesis studies indicated. 22 residues of the PP2Ac C-terminus which are known to be flexible were removed and the remaining structure was docked to TIPRL. The results converged to a model where TIPRL occupies the PP2Ac active site and the spatial arrangement of this complex was compatible with the known position of the PP2Ac C-terminus, which is striking since this information was not used as a restraint. The docking model of TIPRL with PP2Ac residues 1–286 was combined with the position of the C-terminus, obtained from the co-crystal structure of TIPRL with the TEV-derived peptide as shown above. The missing residues 287–309 of PP2Ac were then built using MD free energy perturbation with manual input (Fig. 5A). Compared to the docking models without the C-terminus, addition of the C-terminal residues decreased the free energy and increased the hydrophobic area buried in the interface. Additionally, less conformational variability was present in the ensemble of models containing the C-terminus (Fig. S4)

These results point to an extensive TIPRL-PP2Ac interaction surface which extends from the inner TIPRL cleft where residues 306–309 of PP2Ac are bound, to a second conserved patch on the surface of TIPRL which partially overlaps with the homodimerization surface in the crystal structure reported here, in agreement with results of mutagenesis/pulldown in which point mutations were unable to dissociate the interaction of TIPRL with full length PP2Ac. Superposition of this model on the structure of PP2A trimeric complex indicates that TIPRL partially overlaps with B subunit, but not with PR65/A subunit (Fig. 5B). The TIPRL interaction surface on PP2Ac includes the active site and overlaps with the binding surfaces for LCMT1, PTPA and PME-1 (Fig. 5C,D), supporting a role for TIPRL in PP2A biogenesis or recycling^4^.

TIPRL interacts with the inactive splicing isoform PP2Acα2 reported by us^26^, as well as recombinant PP2Acα expressed in *E. coli*, which may both be analogous to the initial stage of PP2Ac biogenesis represented by the crystal structure of nPP2A-α4 complex^9^. It also interacts with methylated PP2Ac^15^ which corresponds to a later stage of PP2A biogenesis. This raises the intriguing possibility that TIPRL may interact with PP2Ac throughout the biogenesis process, although not simultaneously with PTPA, PME-1 and LCMT-1 due to steric hindrance.

## Discussion

In the present work we described a novel fold for TIPRL which is likely the first of a family including all TIPRL/Tip41 related proteins. The structure reveals the PP2Ac binding site on the surface of TIPRL and allowed its binding specificity to the conserved C-terminus of PP2Ac to be determined. The residues on this binding surface are extremely conserved, as expected for this ancestral function which is shared with the yeast orthologue Tip41.

Previous approaches to map the PP2Ac binding site on TIPRL relied on random mutagenesis^12^ or deletion mapping using a naturally occurring shorter isoform. This short isoform of TIPRL is 178 residues long and comprises residues 1 to 172 of the full length protein together with an additional C-terminal extension of 6 amino acid residues (PGGGHL). Previous studies^27^ reported that this isoform does not interact with PP2Ac and therefore concluded that the binding site is located in the C-terminal half of TIPRL (residues 173–272), which is consistent with our own findings reported here. However, this short isoform may not be correctly folded as its C-terminus is located in strand β7 and the resulting polypeptide thus corresponds to less than a complete domain. Our attempts to express and purify this isoform from *E. coli* resulted in insoluble protein under all conditions tested, suggesting that truncation of the TIPRL sequence at that specific region deeply disturbs it structure (not shown). Not surprisingly, this isoform has never been detected as an endogenous protein.

The insight into the PP2A binding site comes from the unexpected presence of a TEV site peptide bound to TIPRL. If a peptide derived from a widely used sequence such as the TEV site can mimic the C-terminus of PP2Ac, then one would expect to see this happening in other structures of PP2Ac-binding proteins. Indeed, the literature describes a similar case^28^. In the crystal structure of PTPA (PDB ID 4NY3), seven amino acid residues from a TEV-linker were found inside a conserved hydrophobic pocket, which was interpreted as evidence for a protein binding function. However, the authors did not notice the similarity with the PP2A C-terminus. We noticed that the same region of PTPA engages in interaction with the extreme C-terminus of PP2Ac in a crystal structure recently reported^29^ (PDB ID 2G62). It is therefore reasonable to argue that the observed TEV-binding site corresponds to the binding site for the C-terminus of PP2Ac.

We validated the potential peptide-binding cleft on TIPRL by mutating each one of its highly conserved residues and assessing the interaction with PP2Ac by GST-pulldown assays, as well as by measuring direct binding of a synthetic PP2Ac derived peptide to TIPRL by HDX/MS. While no single mutation on TIPRL was able to reduce the interaction with full length PP2Ac, simultaneous mutation of residue R200 together with F180 or I136 showed major effects on its binding to a truncated version of PP2Ac lacking the first 200 residues. The HDX analysis showed that the conformational dynamics changes induced by peptide binding occur mostly on the helical regions on both sites of the proposed peptide binding cleft, but were not restricted to it. Intriguingly, a major conformational change was observed in residues 74–83, suggesting a long range communication between the bottom cleft and helix α2 which is mediated by the connector loop. To a smaller extent, similar results were observed with the phosphorylated peptide. Helix α2 contains the conserved amino acid residue D71 which was shown by our previous studies^12^ to interfere with PP2Ac binding. Due to its position on the opposite side of the peptide binding cleft, helix α2 is unlikely to engage in direct interaction with any part of PP2Ac. Instead, it may function as a regulatory switch, either by engaging in interaction with other proteins, or responding to phosphorylation of nearby residues.

The conservation pattern of the surface adjacent to the peptide binding site indicates a continuity of the PP2A-binding surface from the flexible C-terminus binding site at the C-face cleft of TIPRL to the compact globular domain of the phosphatase which potentially binds to another conserved patch. Interestingly, the docking models pointed to this surface as the site of PP2Ac binding, even without imposing the position of the C-terminus as a restraint. In the crystal structure, this surface is burried in the dimer interface.

The structures of PP2Ac complexed with LCMT1^11^ and PME-1^21^, as well as combined structural information from separate PTPA-containing structures^28^,^29^, show that these proteins bind specifically to the C-terminal tail while engaging in extensive interactions with loop regions surrounding the phosphatase active site. The structural studies shown here indicate that the TIPRL-PP2Ac complex displays features of these biogenesis-related complexes, which supports a potential role of TIPRL in the assembly and recycling of PP2A holoenzyme complexes.

## Methods

### Plasmid construction and mutagenesis

The TIPRL cDNA (acess number NP_690866.1) was amplified from a human fetal brain cDNA library (Clontech) and cloned into pET-TEV, a modified pET28a vector harboring an N-terminal hexa-histidine tag followed by a TEV cleavage site. The TIPRLΔN construct was obtained by amplifying the cDNA from pET-TEV-TIPRL using a forward primer that resulted in a 15-residue N-terminal deletion. The resulting PCR product was subcloned into pET-TEV. The mutants were obtained by thermal cycling with *Pfu* (Thermo Scientific) and digestion of parental DNA with *DpnI* (Thermo Scientific), followed by transformation into DH5α and sequencing. The sequences of primers used for mutagenesis are shown in Table S2.

### Purification and crystallization of TIPRLΔN

His-tagged TIPRLΔN was expressed in *E. coli* BL21(DE3) for 5 hours at 25 °C. Expression was induced with 14 mM lactose. Bacterial cells were resuspended in lysis buffer (Tris buffer 100 mM pH 7.5, 300 mM NaCl, 10 mM β-mercaptoethanol and 5% glycerol supplemented with 1 mM phenylmethylsulfonyl fluoride), incubated with lysozyme (0.1 mg.ml^−1^) for 1 hour at 0 °C and lysed by sonication (10 to 20 cycles of 15 seconds). The cleared lysate obtained after centrifugation (16.000 g, 1 hour at 4 °C) was incubated with Ni Sepharose 6 Fast Flow (GE Healthcare), preequilibrated with Tris buffer 50 mM pH 7.5, 200 mM NaCl, 10 mM β-mercaptoethanol, 5% glycerol and 30 mM imidazole, for 1 hour at 4 °C under slow rotation. The resin was then appied onto an empty chromatography column under gravity flow, washed with buffer (Tris 50 mM pH 7.5, 1 M NaCl, 10 mM β-mercaptoethanol) and bound protein was eluted stepwise with 400 mM and 500 mM imidazole in buffer Tris 50 mM pH 7.5, 200 mM NaCl, 10 mM β-mercaptoethanol and 5% glycerol. The affinity purified protein was cleaved with TEV protease (1:10 mass ratio) for 16 hours at 4 °C and subjected to a final polishing step of size exclusion chromatography on a HiLoad 26/60 Superdex 75 pg column (GE Healthcare) in Tris buffer 20 mM pH 7.5, 100 mM NaCl, 10 mM β-mercaptoethanol. The peak fractions were pooled, the buffer was changed to Tris 20 mM pH 7.5; NaCl 25 mM, β-mercaptoethanol 10 mM using a centrifugal filter device (Millipore, MWCO 10.000) and the sample was concentrated to 70–100 mg.ml^−1^ using the same device. Selenomethionine labelled protein was expressed in minimal medium M9 supplemented with selenomethionine using 0.5 mM IPTG (isopropyl β-D-1-thiogalactopyranoside) as inducer, and purified following essentially the same procedure as the native protein. The initial crystallization attempts were performed in the high throughput crystallization facility of the Brazilian National Biosciences Laboratory, RoboLab. Crystals were grown by vapor diffusion (hanging drop) at 18 °C. Diffraction quality crystals were grown after 10 days in two conditions: 100 mM sodium di-potassium phosphate pH 6 to 6.5, 2 to 3 M NaCl, and 100 mM SPG buffer (Succinic Acid, Sodium Dihydrogen Phosphate and Glycine) pH 5.5–6.7, 2 to 3 M NaCl.

### Structure solution and refinement

Data collection was performed on beamline I02 at the Diamond Light Source/UK. Data processing was done using the Fast_DP pipeline and was followed up by automated phasing with Fast_EP. A strong anomalous signal was present in data sets collected from different crystals. Phasing and initial model building were carried out at 2.66 Å resolution with the data set TIPRL Se-Met (Table 1), as follows. After data analysis and preparation with ShelxC, different ShelxD jobs were run for each of the possible space groups: *P6, P6*_*1*_, *P6*_*2*_ and *P6*_*3*_. For each space group, an automated search was performed for 5, 10, 20, 39 and 78 anomalous sites, which resulted in *P6*_*1*_ as the best space group among those tested up to this step, with 19 anomalous sites found. Next, density modification was done with ShelxE both for *P6*_*1*_ and the enantiomorph *P6*_*5*_, with the solvent fraction varying between 0.25 and 0.75 in 0.05 increments. For all jobs, better FOM and CC values were systematically obtained for the inverted structure, a clear indication that the correct space group was *P6*_*5*_. At this stage, the best statistics resulted from the job in *P6*_*5*_with a solvent fraction of 0.45. Automated model building starting with the latter phase set was then carried out with ARP/wARP and resulted in an initial model where 96% of amino acid residues were modeled with R = 26.12% and R_free_ = 33.10%, corresponding to two molecules in the asymmetric unit and a solvent content of 76.2% as calculated with the program Matthews_Coef (CCP4 package). Further improvement of the model was achieved by visual inspection and manual rebuilding. Datasets were also collected on several additional crystals and data were analysed. One crystal was found to diffract to 2.15 Å when processed using the Xia2/Dials pipeline^30^,^31^,^32^,^33^,^34^,^35^,^36^. This data set was then used for structure solution by molecular replacement with AMoRe (CCP4 package) using the initial model previously obtained. A few residues belonging to turns or loops were built manually. Refinement against the higher resolution data set was done using Coot^37^ and Refmac. Phenix^38^ was used in the final steps and for structure deposition at the Protein Data Bank. Final Ramachandran statistics showed 97.43% residues in favored regions, with R = 18.24% and R_free_ = 19.85%.

### ConSurf analysis of evolutionary conservation

The evolutionary conservation profile of TIPRL was estimated using ConSurf 39,40,41 version 3.0 (consurf.tau.ac.il). A CSI-BLAST search for homologs of the human TIPRL sequence was performed against the UNIREF-90 database with 3 iterations, an E-value cutoff of 0.0001, minimal % ID of 35% for homologs and maximal % ID of 95% between sequences. A total of 150 homologous sequences were retrieved and multiply aligned using MAFFT (with the accurate option -L-INS-i). Calculation of position-specific conservation scores was performed using the Bayesian method. The sequence conservation pattern, color coded by ConSurf from the most variable (turquoise) through intermediately conserved positions (white) to the most conserved (burgundy), was mapped onto the three-dimensional structure of TIPRL and the figures were prepared using PyMol.

### Chemical cross-linking and mass spectrometry

The samples subjected to chemical cross-linking were in 50 mM sodium phosphate buffer, pH 7.5, supplemented with 200 mM NaCl, 5% glycerol (vol/vol) and 10 mM β-mercaptoethanol. Samples were concentrated to 15 mg ml^−1^. The DSS (disuccinimidyl suberate) cross-linker stock solution was 27.1 mM in dimethylformamide and the cross-linking reaction was carried out for two hours at room temperature, using 100x molar excess of DSS to protein sample. The samples were then reduced for 30 minutes at 60 °C with 10 mM DTT (dithiothreitol) in 50 mM ammonium bicarbonate buffer, alkylated for 30 minutes at room temperature using 50 mM iodoacetamide in ammonium bicarbonate buffer and digested with Sequencing Grade Modified Trypsin (Promega) for 17 hours at 37 °C. The resulting peptide mixture was fractionated in five elution steps of 10, 15, 20, 30 and 80% acetonitrile (vol/vol) using Oasis HLB solid phase catridges (Waters Corporation, USA) and loaded onto a nanoAcquity UPLC liquid chromatographer (Waters Corporation, USA) with an Acquity BEH C18 column (100 μm × 100 mm, 1.7 μm) coupled to a Waters Synapt HDMS mass spectrometer (Waters Corporation, USA). 40 pmol of peptide mixture was analyzed in each run. The LC-MS/MS runs were processed on MASCOT Distiller v. 2.4 (Matrix Science Ltd) and analyzed by MASCOT v.2.4. (Matrix Science Ltd). These data obtained from MASCOT Distiller were then analyzed with SIM-XL^42^ to identify the cross-linked peptides.

### Hydrogen-deuterium exchange

Purified, full length TIPRL was incubated for 2 hours with the synthetic tetrapeptide DYFL or DpYFL (tyrosine phosphorylated) at a 1:10 protein:peptide molar ratio in 20 mM pH 7.5 Tris-HCl buffer, 100 mM NaCl, 10 mM β-mercaptoethanol. A control reaction was performed using dimethylformamide instead of the peptides. The hydrogen-deuterium exchange was started by diluting these reactions 1:15 in deuterated buffer at 25 °C and stopped by adding equal volumes of quench buffer (800 mM Guanidine-HCl, 0.8% formic acid (vol/vol), 20 mM DTT 20, pH 2.5) at 4 °C after 0 s (control), 10 s, 1 min, 10 min, 1 h and 2 h. Samples were then injected into a nano Acquity UPLC system with HDX technology coupled to Synapt G1 HDMS (Waters Corporation, USA). Online digestion was performed on an immobilized pepsin column (2 × 30 mm, Applied Biosystem, USA) for 3 minutes at 15 °C with 35 μL.min^−1^ flow. The resulting peptides were desalted on an ACQUITY UPLC BEH C18 pre-column (1,7 μm, VanGuard, Waters) at 0 °C and separated on an analytical column (ACQUITY UPLC BEH C18 1,7 μm, 1 mm × 100 mm, Waters) at 0 °C with 50 μL.min-1 flow. The runs were processed by Protein Lynx Global Server v.2.4. (Waters Corporation, USA) and DynamX v.3.0 (Waters Corporation, USA).

### GST pulldown assays

The expression plasmids for His-tagged, full length TIPRL and GST-tagged PP2Ac alpha, or the respective mutated constructs were cotransformed into *E. coli* BL21(DE3) and coexpression was induced by adding 0.5 mM IPTG and incubating for 5 hours at 25 °C. The pellets were lysed using 0.1 mg ml^−1^ lysozyme combined with sonication in pH 7.4 PBS buffer supplemented with 0.5% (v/v) IGEPAL CA-630, 1 mM DTT and 0.1 mM phenylmethylsulfonyl fluoride. Glutathione sepharose beads (Glutathione-Sepharose 4B, GE Healthcare) were added to the cleared lysates, which were incubated for one hour at 4 °C. The samples were washed three times with lysis buffer and the beads were eluted with sample buffer and analyzed by coomassie stained SDS-PAGE.

### Docking

All computational calculations were performed employing protocols included in Rosetta Molecular Modeling Suite^43^. Crystallographic structures of three complexes containing PP2Ac with PTPA, LCMT-1and PME-1 (PDB ID: 4LAC, 3P71 and 3C5W, respectively) indicate that C-terminus of PP2Ac is flexible, adopting a conformation responsible for a direct interaction with its partner. The most complete construction available of PP2Ac (PDB ID: 3P71, chain C) was selected as a starting structure for our protocol, in which the flexible C-terminus was manually removed. To prepare the structures for docking and pre sample conformational diversity, the starting structures of PP2A and TIPRL were relaxed to generate 100 candidate structures. The four lowest score candidates of each individual protein were employed to manually build 16 complex structure proposals in PyMol^44^. The initial relative orientation between PP2Ac and TIPRL was build based on the presence of the C-terminus peptide of PP2Ac in the crystal structure of TIPRL, which allowed to reduce the sampling space. We applied RosettaDock protocol to generate 250 models for each initial candidate totalizing 4000 docking structural models. The 10 best models evaluated by the binding energy associated to the interface were selected to another step of relax to produce 200 decoys. Finally, the best model was employed to iterative steps of manual reconstruction of the C-terminus of PP2A and relax protocol application. Additionally, crystallographic structures of three complexes of PP2A (PDB: 4LAC; 3P71 and 3C5W) were relaxed to generate 10 decoys each and evaluated by the same interface score metrics and served as a quality check of the interface metrics of the final model of the interaction between PP2Ac and TIPRL.

## Additional Information

**Accession codes:** The atomic coordinates have been deposited in the Protein Data Bank under accession code 5D9G.

**How to cite this article**: Scorsato, V. *et al*. Crystal structure of the human Tip41 orthologue, TIPRL, reveals a novel fold and a binding site for the PP2Ac C-terminus. *Sci. Rep.* **6**, 30813; doi: 10.1038/srep30813 (2016).

## Supplementary Material

Supplementary Information

Supplementary Information

## Figures and Tables

**Table 1:** Data collection and statistics Values in parentheses refer to the last resolution shell.

Data Collection	TIPRL Se-Met	TIPRL
*Data collection statistics*		
Space Group	*P6*_5_	*P6*_5_
Cell dimensions (Å) *a, b, c.*	143.83, 145.83, 95.93	145.31, 145.31, 95.95
Detector	PILATUS 6M	PILATUS 6M
X-ray source	DLS I02	DLS I02
Wavelength (Å)	0.9922	0.9922
Resolution range (Å)	29.29–2.66	95.95–2.15
Last resolution shell (Å)	2.73–2.66	2.25–2.15
Wilson Plot B-factor (Å^2^)	69.29	55.45
Redundancy	6.1 (5.4)	15.2 (15.1)
*Rpim* (%)	4.7 (35.5)	3.1 (41.3)
CC (1/2)	0.996 (0.661)	0.995 (0.760)
Completeness(%)	99.0 (97.4)	100.0 (100.0)
Total reflections	200998 (12787)	952365 (125665)
Unique reflections	33078 (2388)	62695 (8301)
I/σ(I)	12.5 (1.9)	12.1 (2.3)
ΔAnom correlation between half sets	0.584 (0.076)	*
Mid-Slope of Anom Normal Probability	1.314	0.927
*Refinement statistics*		
Reflections used for refinement		62515 (3277**)
*R* (%)		18.24
*R*_Free_(%)		19.85
No. of protein atoms		3969
No. of water molecules		233
No. of peptide atoms		99
Average B-factor (Å^2^)		
Overall		58.60
Protein atoms		58.13
Water molecules		58,32
Peptide atoms		71,61
All-atom Clash score		4.71
Coordinate Error (ML based) (Å)		0.30
Phase error (°)		27.89
RMS deviations from ideality		
RMS bond lengths (Å)		0.003
RMS bond angles (°)		0.691
*Ramachandran analysis*		
Favored (%)		97.43
Allowed (%)		2.17
Outliers (%)		0.40
PDB ID		5D9G

Wilson Plot B-factors were calculated with SFCheck^45^. Average B-factors include TLS contribution.

^*^No significant anomalous signal.

^**^Shell 2.19–2.15 Å.
