# Supplementary material for: Crystal structure of the human Tip41 orthologue, TIPRL, reveals a novel fold and a binding site for the PP2Ac C-terminus
Source: Sci Rep. 2016 Aug 4;6:30813. doi: 10.1038/srep30813 (PMC4973239; doi:10.1038/srep30813)

Supplemental Information

Title: Crystal structure of the human Tip41 orthologue, TIPRL, reveals a novel fold and a binding site for the PP2Ac C-terminus

Authors:

Valéria Scorsato[1, 2, 3,] Tatiani B. Lima[1,2], Germanna L. Righetto[2], Nilson I. T. Zanchin[4], José Brandão-Neto[5], James Sandy[5], Humberto D'Muniz Pereira[6], Állan Ferrari[1], Fabio C. Gozzo[1], Juliana H. C. Smetana[2*] and Ricardo Aparicio[1*]

Affiliations:

1. Institute of Chemistry, University of Campinas, Campinas, São Paulo 13083-970, Brazil.

2. Brazilian National Laboratory for Biosciences, Center for Research in Energy and Materials, Campinas, São Paulo 13084-971, Brazil.

3. Institute of Biology, University of Campinas, Campinas, São Paulo 13083-970, Brazil.

4. Carlos Chagas Insitute, Oswaldo Cruz Foundation (FIOCRUZ), Curitiba, PR, Brazil,

5. Diamond Light Source, OX11 0DE, Chilton, UK

6. Physics Institute of São Carlos, Brazil

* Co-senior author.

Correspondence and requests for materials should be addressed to J.H.C.S (e-mail juliana.smetana@lnbio.cnpem.br) or R.A. (e-mail aparicio@iqm.unicamp.br).

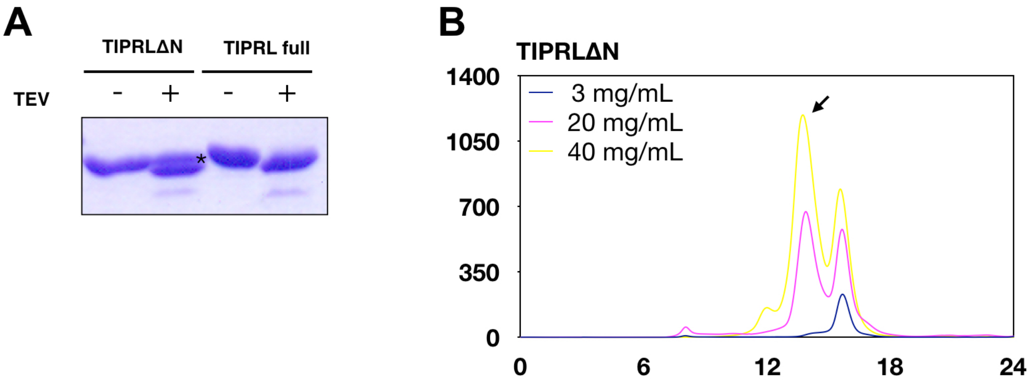

Fig. S1. Dimerization of TIPRLΔN after TEV cleavage.

(A) Coomassie stained SDS-PAGE analysis of purified TIPRLΔN and full length TIPRL, before (-) and after (+) removal of the histidine tag with TEV protease. TIPRLΔN undergoes partial cleavage as seen by the presence of a double band, while full length TIPRL is completely cleaved under the same conditions. Uncleaved protein in the second lane is marked with an asterisk. TEV protease appears as a faint band in the second and fourth lanes. (B) Size exclusion profiles of TIPRLΔN on a Superdex 200 10/30 column after cleavage with the TEV protease. TIPRLΔN was analyzed at three different concentrations - 3, 20 and 40 mg ml^{-1}, showing a concentration-dependent dimerization which was observed as a second peak eluting around 14 ml, indicated with a black arrow. Uncleaved TIPRLΔN, as well as full length TIPRL both cleaved and uncleaved (not shown) were not able to form this second peak under similar conditions.

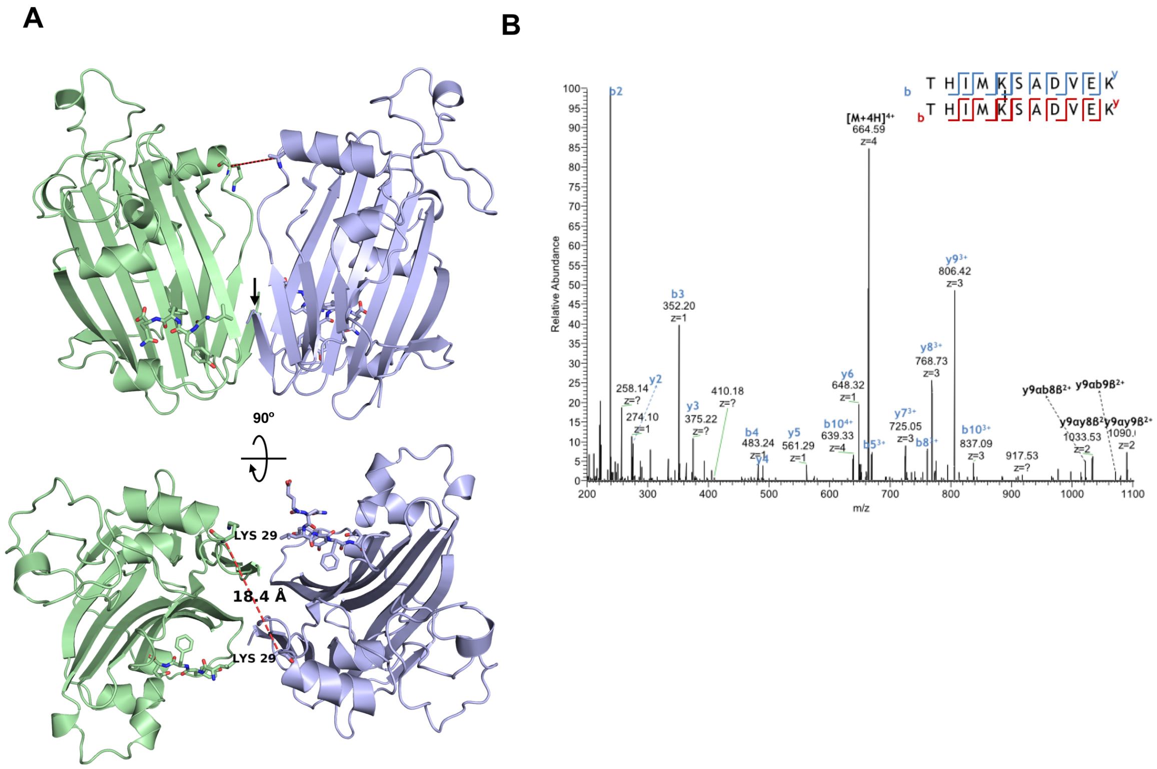

Fig. S2. Cross-linking/MS analysis of TIPRLΔN dimerization. (A) The crystal structure of TIPRLΔN contains two molecules in the asymmetric unit that seem to form a dimer, depicted as pale green (chain A) and blue (chain B) cartoon representations. The dimer interface involves the N-terminus, more specifically the β1 strand. Cross-linking-MS analysis detected an interpeptide crosslink between Lys29 in both molecules, suggesting that this N-terminal dimer is also observed in solution. Lys29 is shown in sticks representation and the 18.4Å distance to its counterpart in the other molecule is indicated with a red trace. The black arrow indicates the position of the N-terminus. The bound peptides are shown in sticks representation. (B) MS spectrum of the detected cross-link.

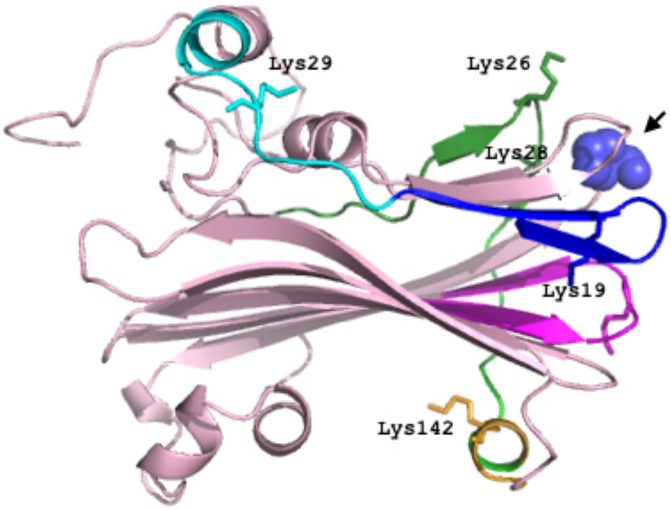


```
  1 MMIHGFQSSH RDFCFGPWKL TASKTHIMKS ADVEKLADEL HMPSLPEMMF
 51 GDNVLRIQHG SGFGIEFNAT DALRCVNNYQ GMLKVACAEE WQESRTEGEH
101 SKEVIKPYDW TYTTDYKGTL LGESLKLKVV PTTDHIDTEK LKAREQIKFF
151 EEVLLFEDEL HDHGVSSLSV KIRVMPSSFF LLLRFFLRID GVLIRMNDTR
201 LYHEADKTYM LREYTSRESK ISSLMHVPPS LFTEPNEISQ YLPIKEAVCE
251 KLIFPERIDP NPADSQKSTQ VE
```

Figure S3. Crosslinking/MS analysis of the C-terminus of TIPRL. The C-terminal lysine 267 was crosslinked to each one of the tryptic peptides which are highlighted in different colors on the TIPRL structure (top) and sequence (bottom). Each cross-linked lysine is shown as sticks in the structure and highlighted in bold in the sequence. The arrow indicated the position of the C-terminus. The tryptic peptide shown in red does not appear in the electron density.

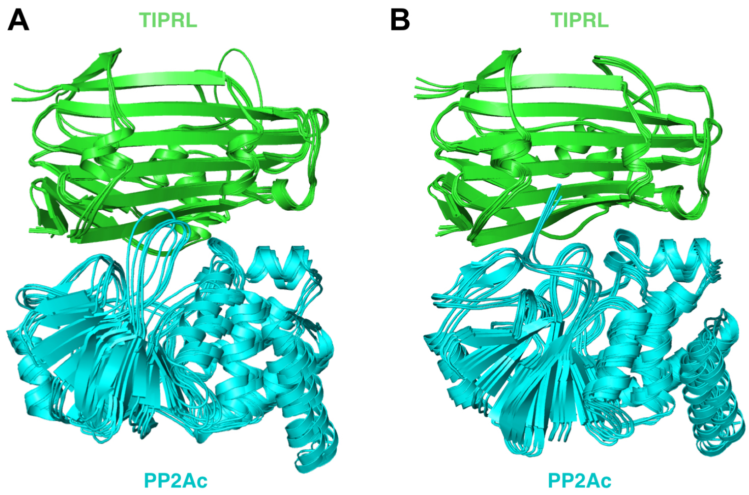

Figure S4. Docking models of the TIPRL-PP2Ac interaction without the C-terminal region of PP2Ac (A) and after insertion of the C-terminus with manual building of the connecting residues (B). An ensemble of five models is shown in each panel.

Table S1. TIPRL DSS cross-linked inter-peptides (labeled alpha and beta) and intra-peptides identified by LC-MS/MS. Cross-linked residues are underlined.

Cross-linked inter-peptides	
Peptide alpha	Peptide beta
LKVVPTTDHIDTEK	LKAR
LYHEADKTYMLR	LKAR
IDPNPADSQKSTQVE	LKAR
THIMKSADVEK	LKAR
IDPNPADSQKSTQVE	THIMKSADVEK
LYHEADKTYMLR	THIMKSADVEK
IDPNPADSQKSTQVE	LKVVPTTDHIDTEK
EAVCEKLIFPER	LKAR
LKVVPTTDHIDTEK	LYHEADKTYMLR
IDPNPADSQKSTQVE	LYHEADKTYMLR
IDPNPADSQKSTQVE	GTLLGESLKLK
IDPNPADSQKSTQVE	EAVCEKLIFPER
LYHEADKTYMLR	EAVCEKLIFPER
THIMKSADVEK	THIMKSADVEK
ESKISSLMHVPPSLFTEPNEISQYLPIK	EVIKPYDWTYTTDYK
ESKISSLMHVPPSLFTEPNEISQYLPIK	CVNNYQGMLKVACAEEWQESR
SADVEKLADELHMPSLPEMMFGDNVLR	IDPNPADSQKSTQVE
LKVVPTTDHIDTEK	EAVCEKLIFPER
VVPTTDHIDTEKLK	LYHEADKTYMLR
ESKISSLMHVPPSLFTEPNEISQYLPIK	EAVCEKLIFPER
TEGEHSKEVIKPYDWTYTTDYK	GTLLGESLKLK
TEGEHSKEVIKPYDWTYTTDYK	LYHEADKTYMLR
IDPNPADSQKSTQVE	DFCFGPWKLTASK

Cross-linked intra-peptides

THIMKSADVEKLADELHMPSLPEMMFGDNVLR
ESKISSLMHVPPSLFTEPNEISQYLPIKEAVCEK
EVIKPYDWTYTTDYKGTLLGESLK
GTLLGESLKLKVVPTTDHIDTEK
ISSLMHVPPSLFTEPNEISQYLPIKEAVCEKLIFPER
LTASKTHIMKSADVEK
TEGEHSKEVIKPYDWTYTTDYK
VVPTTDHIDTEKLKAR
ESKISSLMHVPPSLFTEPNEISQYLPIK

Table S2. Sequences of mutagenic primers

Mutants	Sequence (5' - 3')
**TIPRL**	
N68A-F	catcttaacgcatctgtagcagcgaactcaattccaaagccaga
N68A-R	tctggctttggaattgagttcgctgctacagatgcgttaagatg
D71L-F	gttgtttacacatcttaacgctaatgtagcattgaactcaattccaaagccaga
D71L-R	tctggctttggaattgagttcaatgctacattagcgttaagatgtgtaaacaac
I136T-F	ctctggctttcaatttttctgtatccgtatgatctgttgtaggtacaacctt
I136T-R	aaggttgtacctacaacagatcatacggatacagaaaaattgaaagccagag
L141A-F	cttaatctgttctctggctttcgctttttctgtatctatatgatctgttgtag
L141A-R	ctacaacagatcatatagatacagaaaaagcgaaagccagagaacagattaag
I147A-F	aaaggagaacttcttcaaaaaacttagcctgttctctggctttcaatttttctg
I147A-R	cagaaaaattgaaagccagagaacaggctaagttttttgaagaagttctccttt
F150A-F	aaaaggagaacttcttcagcaaacttaatctgttctctggctttcaatttttctg
F150A-R	cagaaaaattgaaagccagagaacagattaagtttgctgaagaagttctcctttt
F180A-F	ccgcaacagcagggcaaagctagaaggcattactctaatc
F180A-R	gattagagtaatgccttctagctttgccctgctgttgcgg
L182A-F	ctcaagaaaaaccgcaacgccaggaaaaagctagaaggca
L182A-R	tgccttctagctttttcctggcgttgcggtttttcttgag
R184A-F	ccatcaattctcaagaaaaacgccaacagcaggaaaaagctaga
R184A-R	tctagctttttcctgctgttggcgtttttcttgagaattgatgg
R200A-F	ttgtcagcctcatggtaaagtgccgtgtcattcattctgataag
R200A-R	cttatcagaatgaatgacacggcactttaccatgaggctgacaa
S239E-F	cttcctttattggtaaatactgctctatttcattaggttccgtgaagagggaag
S239E-R	cttccctcttcacggaacctaatgaaatagagcagtatttaccaataaaggaag
F180L182AA-F	tctcaagaaaaaccgcaacgccagggcaaagctagaaggcattactctaatcttcaca
F180L182AA-R	tgtgaagattagagtaatgccttctagctttgccctggcgttgcggtttttcttgaga
**PP2Ac**	
C-Terminal	aggagaaatttataggaactcgtctggggtgcgccgtg
C-Terminal	cacggcgcaccccagacgagttcctataaatttctcct
C-Terminal	aatttataggaagtagtctggctagcgccgtgtaacatgaggctc
C-Terminal	gagcctcatgttacacggcgctagccagactacttcctataaatt
PP2Acβ _Y307E_F	attggatccttacaggaactcgtctggggtacgacgag
PP2Acβ_Y307E_R	ctcgtcgtaccccagacgagttcctgtaaggatccaat
PP2Acβ_T304Stop	cttacaggaagtagtctggctaacgacgagtaacatgtggctc
PP2Acβ_T304Stop	gagccacatgttactcgtcgttagccagactacttcctgtaag

SUPPLEMENTAL EXPERIMENTAL PROCEDURES

Docking. All computational calculations were performed employing protocols included in Rosetta Molecular Modeling Suite (rosetta_src_2015.38.58158_bundle version): Relax protocol (relax.mpi.linuxccrelease), RosettaDock protocol (docking_protocol.mpi.linuxccrelease), Interface Analyser application (Interface_Analyser.mpi.linuxccrelease)**.**
The list of options for each protocol is described below.

```
# Relax protocol
-database {Rosetta database directory}
-l {file containing a list of pdb files name to process}
-ex1
-ex2
-use_input_sc
-out:file:scorefile (output file name desired)
-out:prefix (desired prefix to add to each decoy)
-nstruct (number of decoys to generate)

# RosettaDock protocol
-database {Rosetta database directory}
-l {file containing a list of pdb files name to process}
-dock_pert 3 8
-ex1
-ex2aro
-use_input_sc
-out:file:scorefile (output file name desired)
-out:prefix (desired prefix to add to each decoy)
-nstruct (number of decoys to generate)

#Interface Analyser application
-database {Rosetta database directory}
-l {file containing a list of pdb files name to process}
-compute_packstat
-ex1
-ex2aro
-use_input_sc
-pack_input
-pack_separated
-add_regular_score_to_scorefile
-out:file:scorefile (output file name desired)
-out:file:score_only
```

Supplementary Dataset 1. TIPRL Cross-linking/MS spectra. Related to Fig. S2 and Table S1.

Inter-peptide 1 (TIPRLΔN)

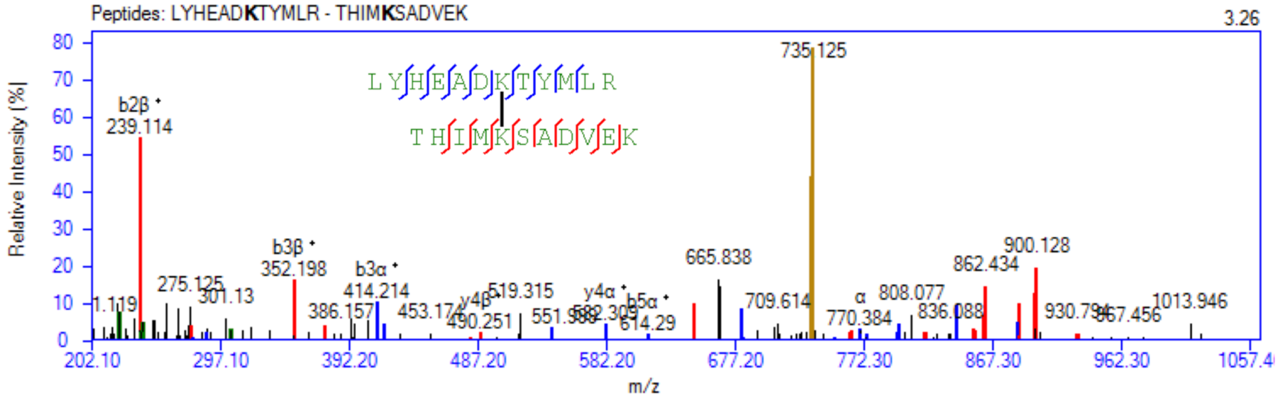

Inter-peptide 2 (TIPRLΔN)

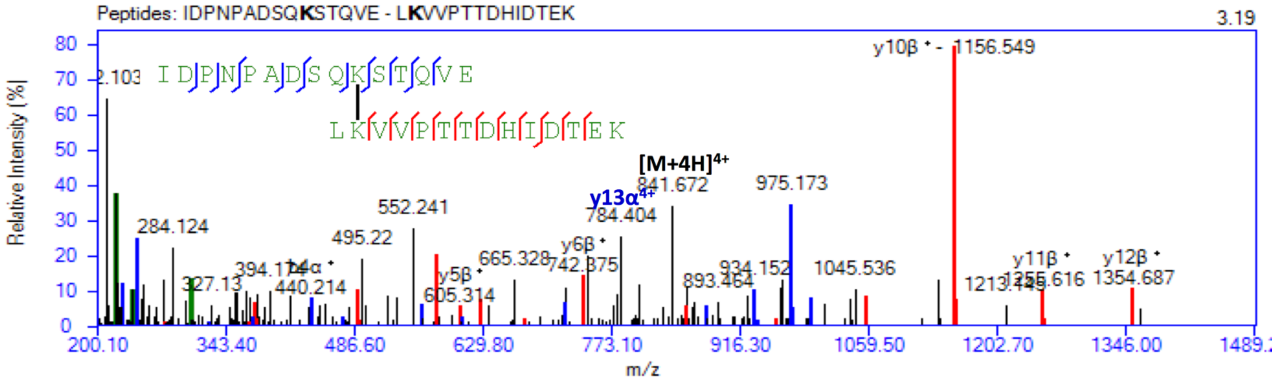

Inter-peptide 3 (TIPRLΔN)

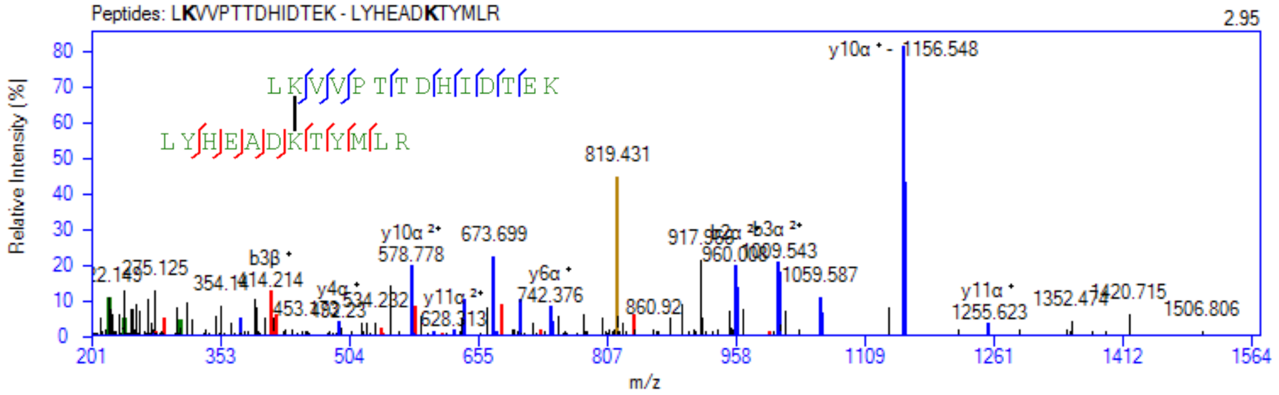

Inter-peptide 4 (TIPRLΔN)

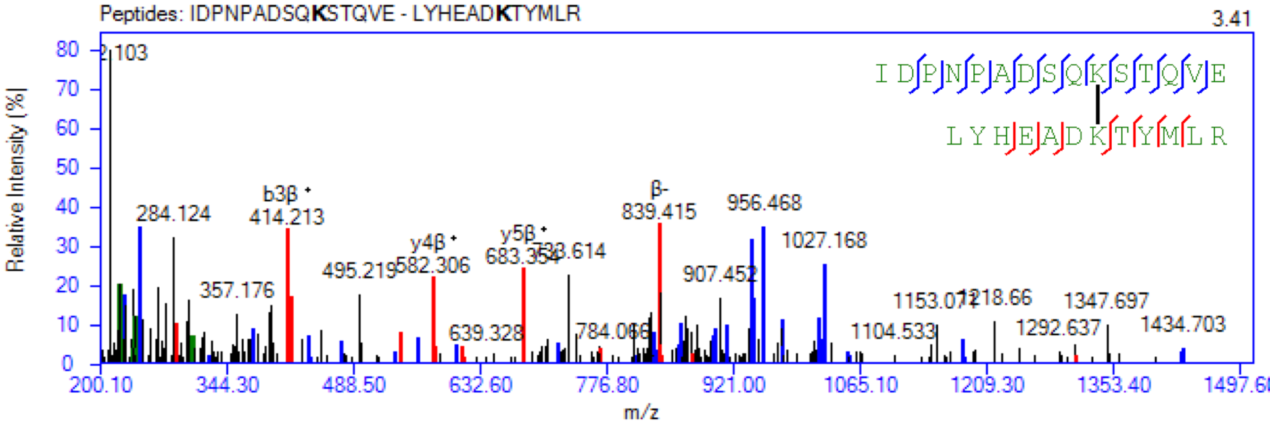

Inter-peptide 5 (TIPRLΔN)

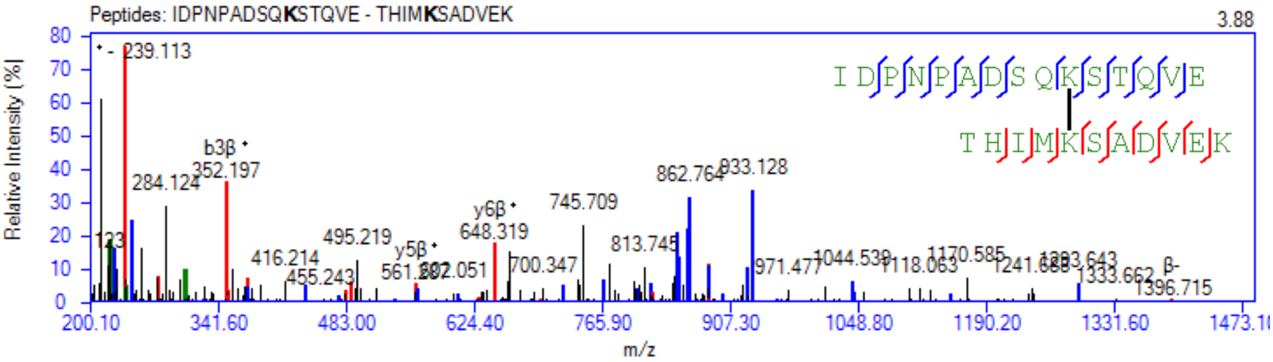

Inter-peptide 6 (TIPRLΔN)

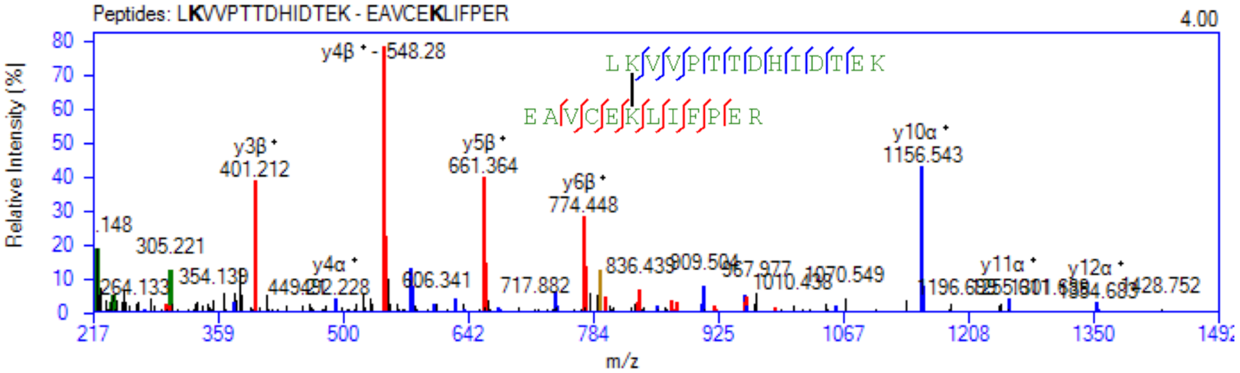

Inter-peptide 7 (TIPRLΔN)

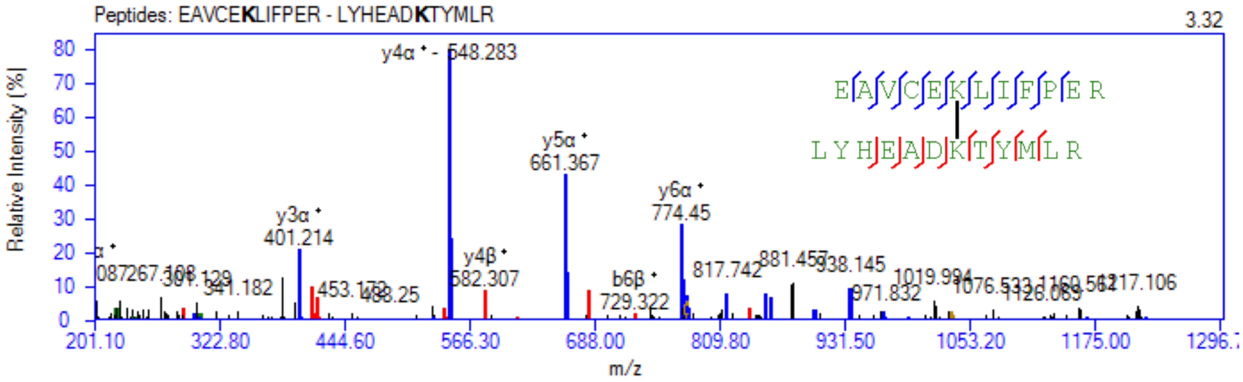

Inter-peptide 8 (TIPRLΔN)

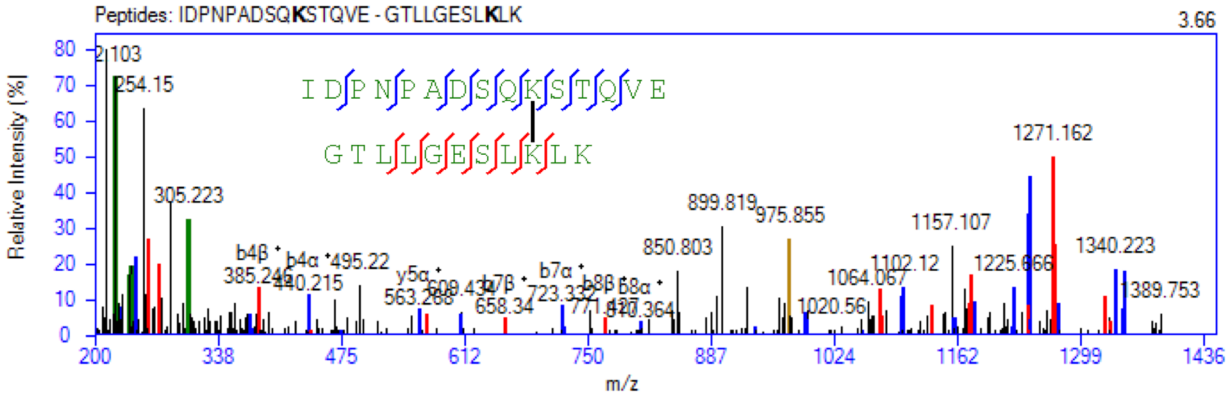

Intra-peptide 1 (TIPRLΔN)

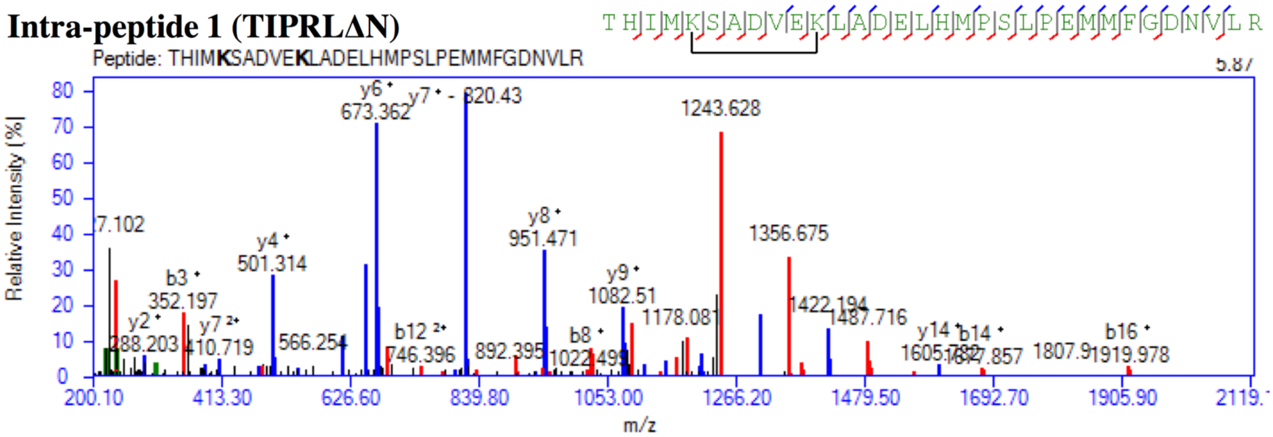

Intra-peptide 2 (TIPRLΔN)

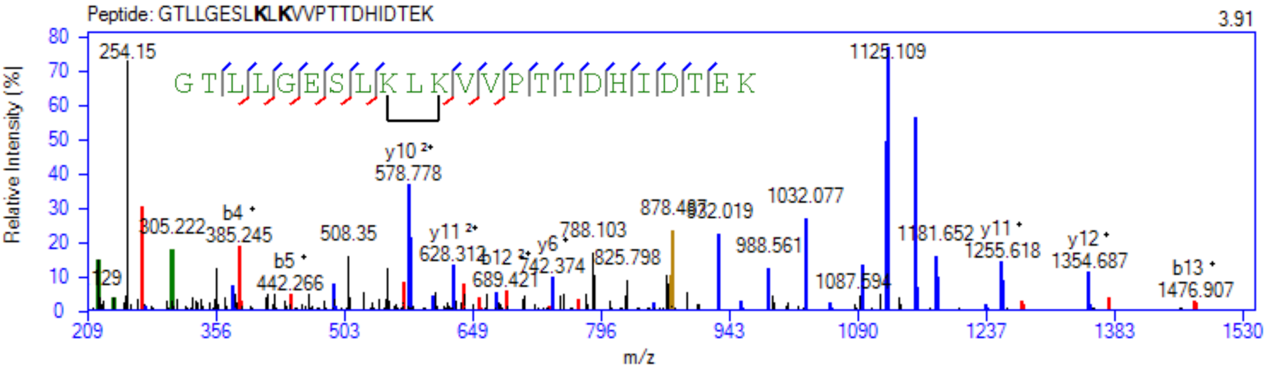

Intra-peptide 3 (TIPRLΔN)

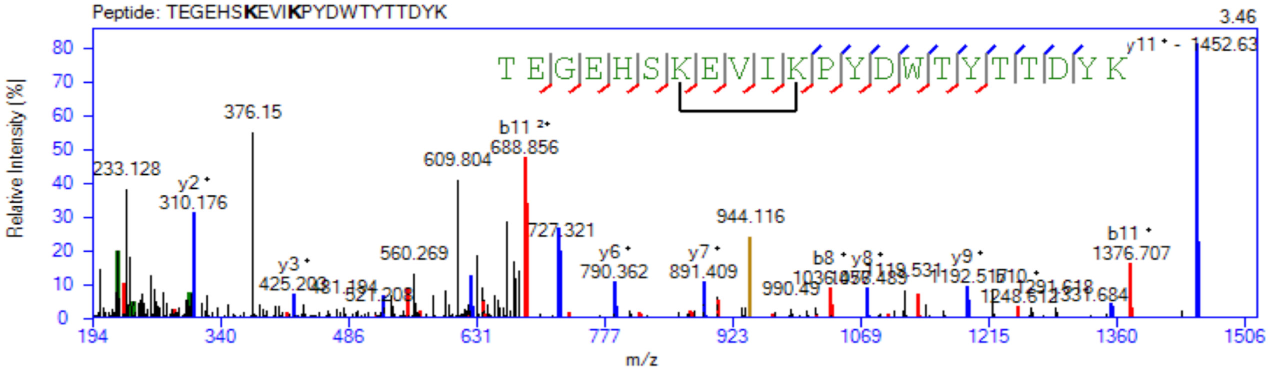

Intra-peptide 4 (TIPRLΔN)

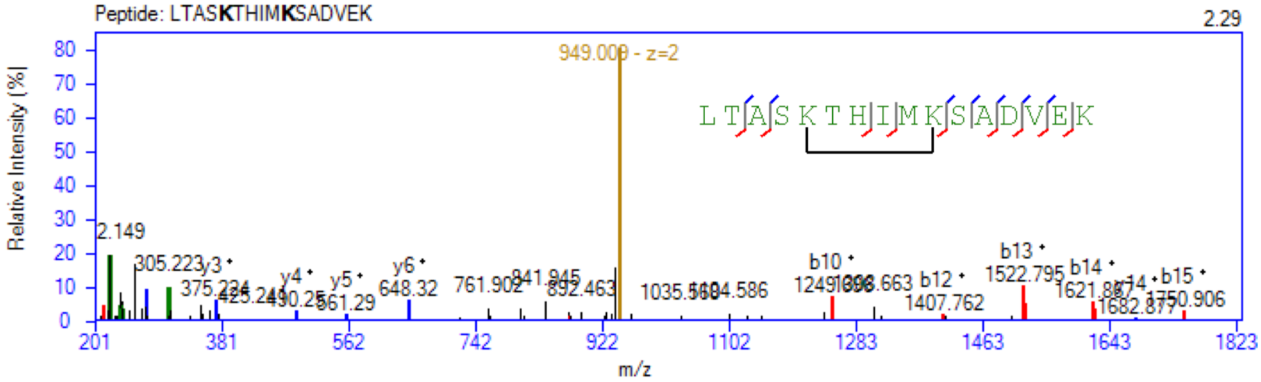

Supplementary Dataset 2. Sequence coverage map and deuterium incorporation plots related to Fig. 4 d, e, f.

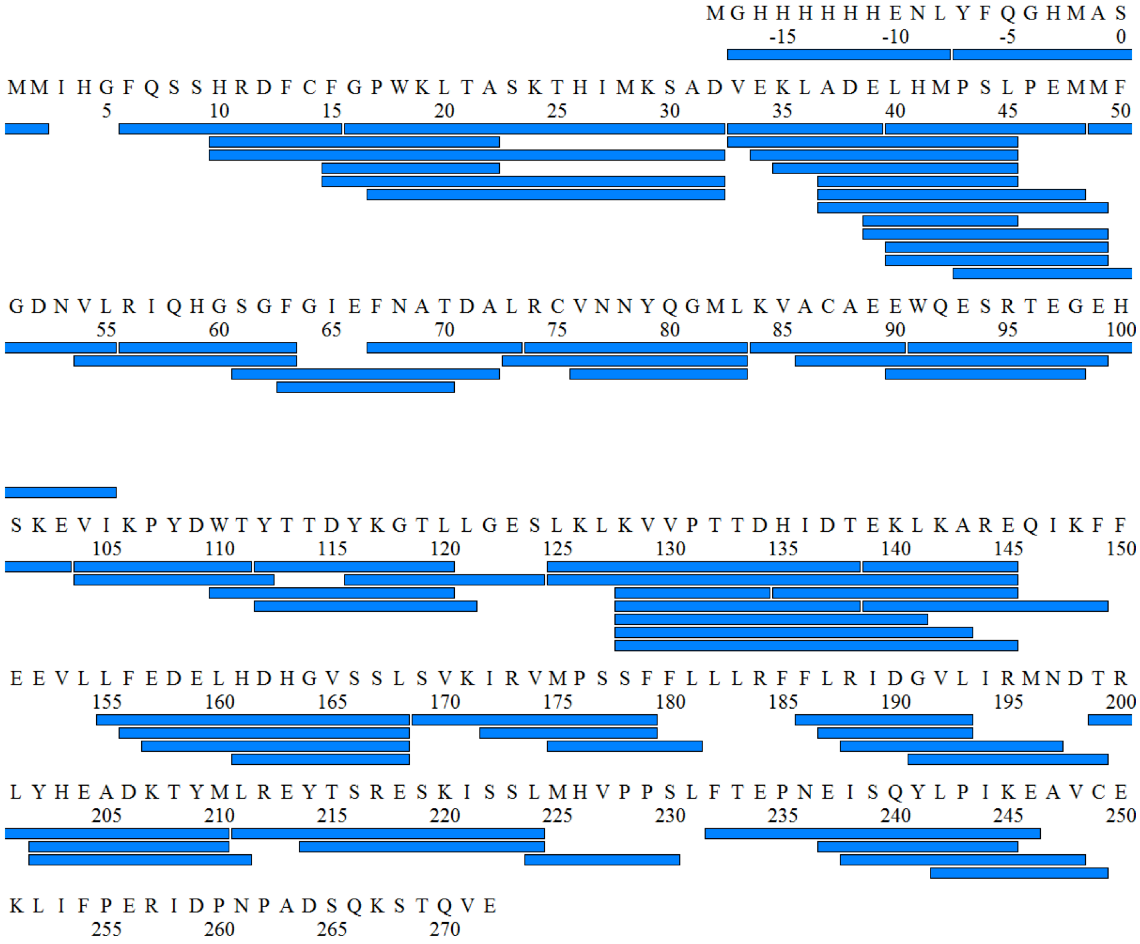

Total: 72 Peptides, 87.3% Coverage, 3.09 Redundancy

Sequence coverage map for TIPRL. Solid blue bar under the sequence denotes the peptides identified after HDX-MS analysis with approximately 87% of sequence coverage.

Deuterium incorporation plots related to Fig. 4 d, e, f.

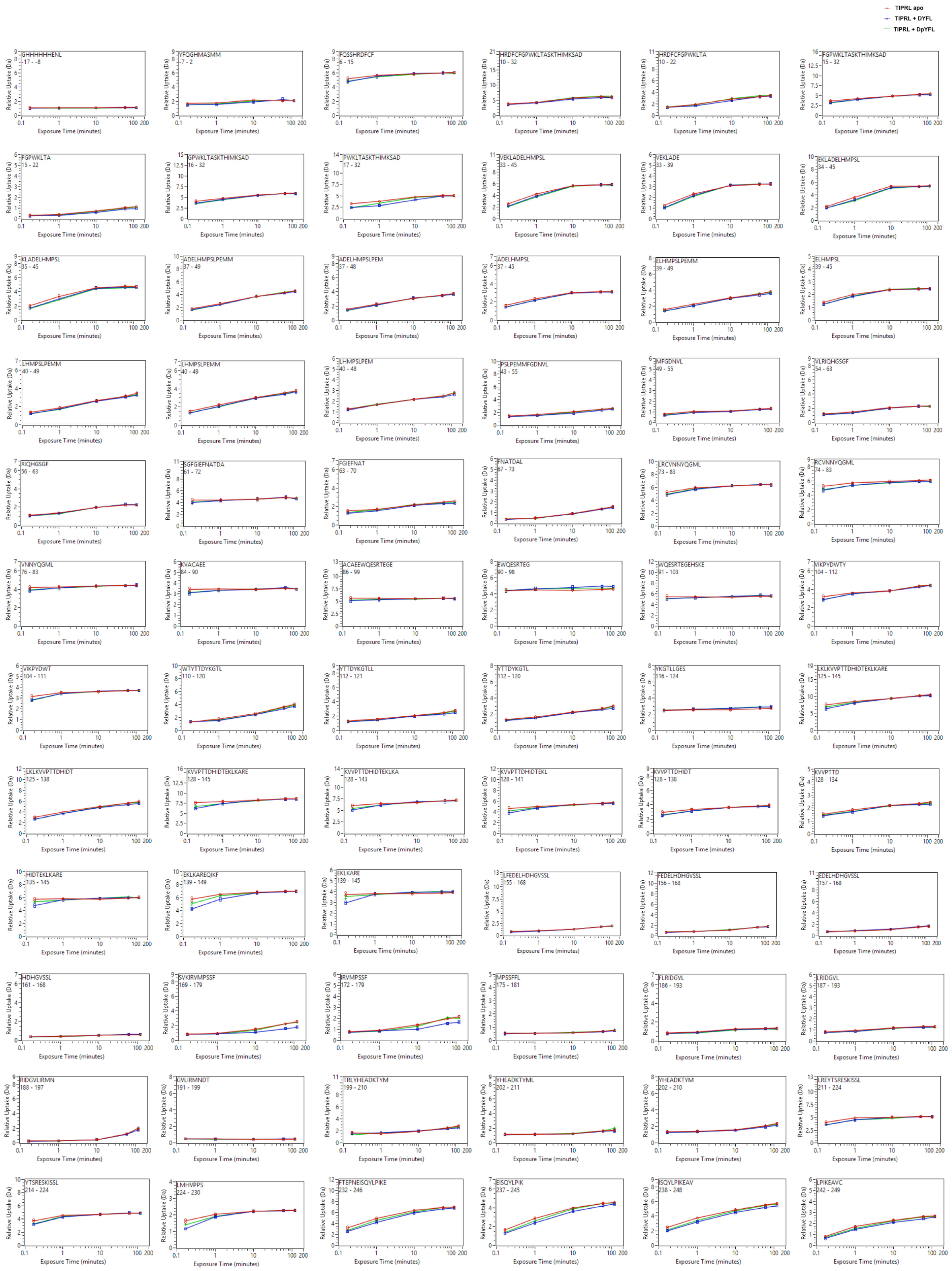

Supplement: Supplementary Information [file srep30813-s1.pdf]
